# Supplementary material for: In vitro culture of leukemic cells in collagen scaffolds and carboxymethyl cellulose-polyethylene glycol gel
Source: PeerJ. 2024 Dec 6;12:e18637. doi: 10.7717/peerj.18637 (PMC11627079; doi:10.7717/peerj.18637)
Supplement: Supplemental Information 1 — The templates were following: human RNA (10 ng per reaction), murine RNA (100 ng per reaction), RNA from an unseeded collagen scaffold, RNA from an unseeded CMC-PEG gel, negative control (nuclease-free water). ΔRn – difference between normalized reporter (Rn) at the end point and at the starting point. [file peerj-12-18637-s001.pdf]

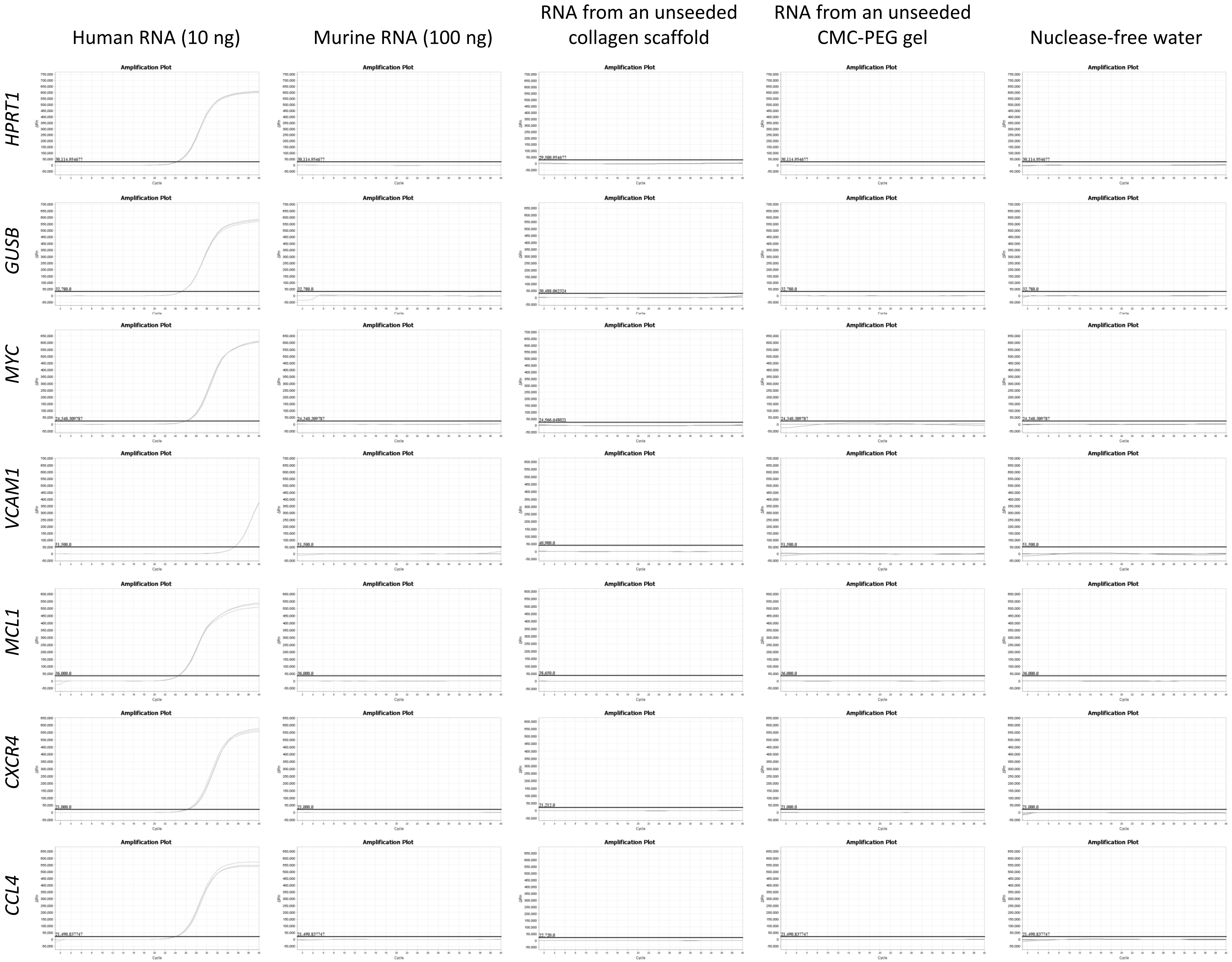
Human RNA (10 ng)
Murine RNA (100 ng)
RNA from an unseeded collagen scaffold
RNA from an unseeded CMC-PEG gel
Nuclease-free water
HPRT1
GUSB
MYC
VCAM1
MCL1
CXCR4
CCL4
Amplification Plot
Cycle
ΔRn
