# Supplementary figures and images for: In vitro culture of leukemic cells in collagen scaffolds and carboxymethyl cellulose-polyethylene glycol gel
Source: PeerJ. 2024 Dec 6;12:e18637. doi: 10.7717/peerj.18637 (PMC11627079; doi:10.7717/peerj.18637)

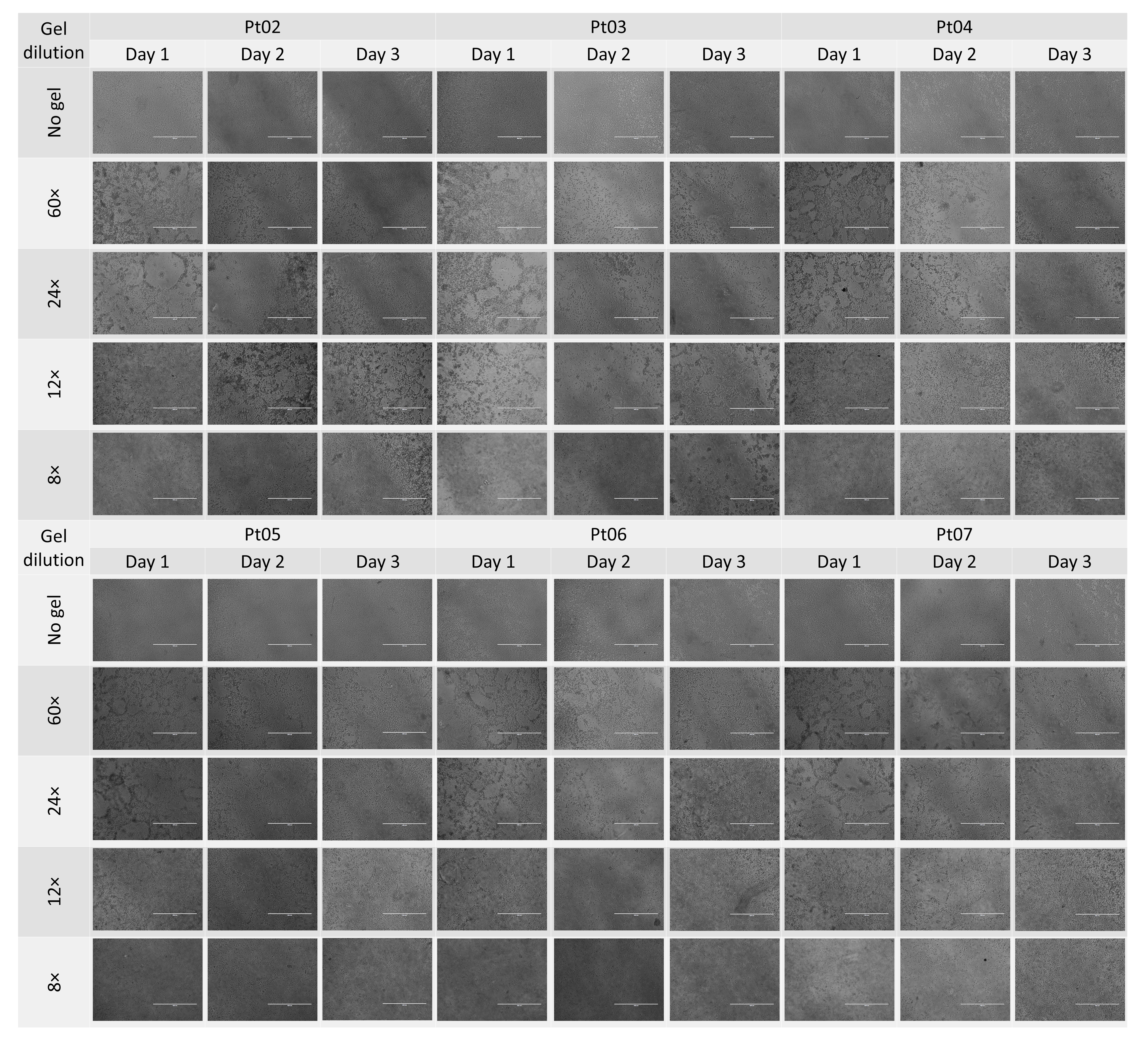

Supplement: Supplemental Information 2 — Cells were cultured conventionally (no gel) and in several dilutions (8×, 12×, 24×, 60×) of CMC-PEG gel for 3 days. Captured with EVOS FL microscope, transmission channel. Scale bar denotes 400 μm. [file peerj-12-18637-s002.png]

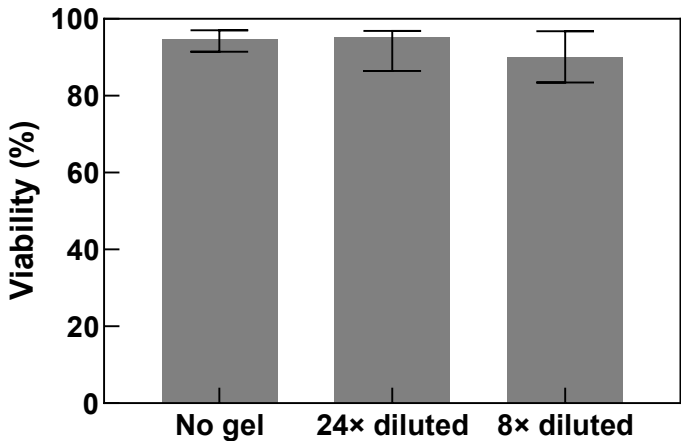

Supplement: Supplemental Information 3 — No gel – median 94.73% (min 91.42%, max 97.00%), 24× diluted gel – median 95.29% (min 86.43%, max 96.84%), 8× diluted gel – median 89.92 % (min 83.48%, max 96.78 %). N=6 biological replicates. The calculations can be seen in the accompanying raw data files (https://doi.org/10.5281/zenodo.13933534). <!--[if !supportAnnotations]--> <!--[endif]--> [file peerj-12-18637-s003.pdf]

**MYC**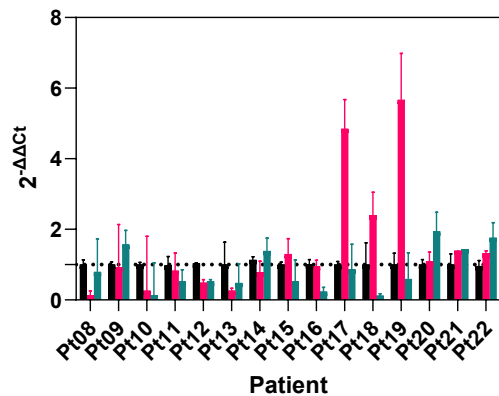**VCAM1**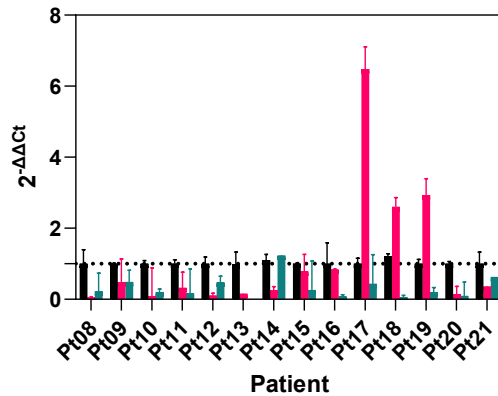**MCL1**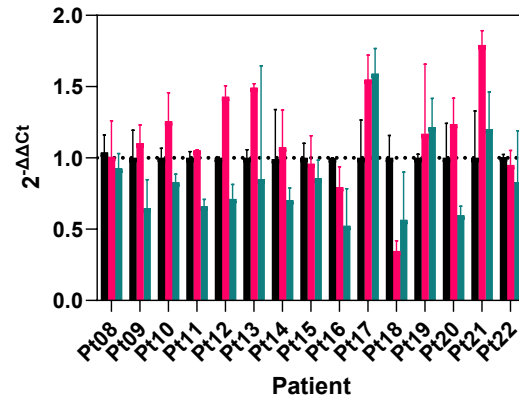**CXCR4**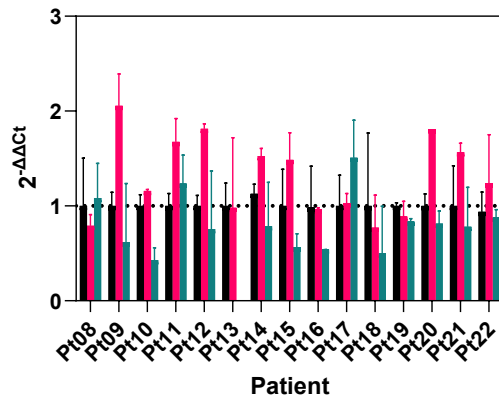**CCL4**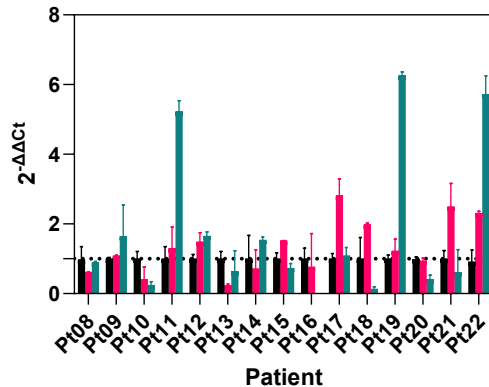

■ Conventional  
■ CMC-PEG gel  
■ Collagen scaffold

Supplement: Supplemental Information 5 — N=3 biological replicates for each culture. [file peerj-12-18637-s005.pdf]
