# Supplementary material for: In vitro culture of leukemic cells in collagen scaffolds and carboxymethyl cellulose-polyethylene glycol gel
Source: PeerJ. 2024 Dec 6;12:e18637. doi: 10.7717/peerj.18637 (PMC11627079; doi:10.7717/peerj.18637)
Supplement: Supplemental Information 4 — Measured by AlamarBlue, CMC-PEG gel stock dilution 24× was used. Unmut – unmutated, mut – mutated, wt – wild type, RFU – Relative Fluorescence Units, i.v. – initial viability. N=3 biological replicates for each culture. [file peerj-12-18637-s004.pdf]

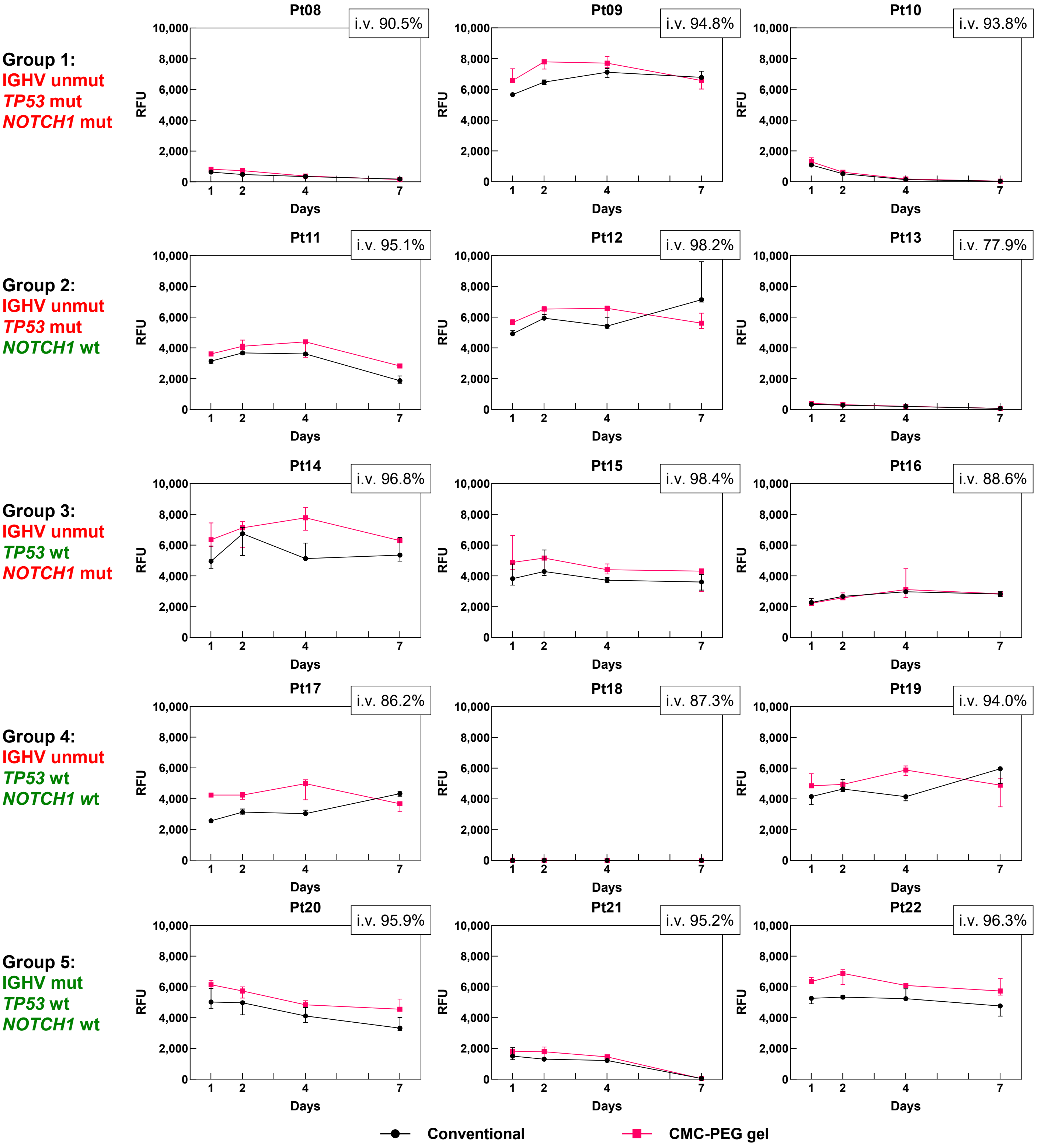
Group 1:
IGHV unmut
TP53 mut
NOTCH1 mut
Pt08
i.v. 90.5%
Pt09
i.v. 94.8%
Pt10
i.v. 93.8%
Group 2:
IGHV unmut
TP53 mut
NOTCH1 wt
Pt11
i.v. 95.1%
Pt12
i.v. 98.2%
Pt13
i.v. 77.9%
Group 3:
IGHV unmut
TP53 wt
NOTCH1 mut
Pt14
i.v. 96.8%
Pt15
i.v. 98.4%
Pt16
i.v. 88.6%
Group 4:
IGHV unmut
TP53 wt
NOTCH1 wt
Pt17
i.v. 86.2%
Pt18
i.v. 87.3%
Pt19
i.v. 94.0%
Group 5:
IGHV mut
TP53 wt
NOTCH1 wt
Pt20
i.v. 95.9%
Pt21
i.v. 95.2%
Pt22
i.v. 96.3%
RFU
0
2,000
4,000
6,000
8,000
10,000
Days
1
2
4
7
Conventional
CMC-PEG gel
