# Supplementary material for: In vitro culture of leukemic cells in collagen scaffolds and carboxymethyl cellulose-polyethylene glycol gel
Source: PeerJ. 2024 Dec 6;12:e18637. doi: 10.7717/peerj.18637 (PMC11627079; doi:10.7717/peerj.18637)
Supplement: Supplemental Information 6 [file peerj-12-18637-s006.docx]

**Description of RT-qPCR according to the MIQE guidelines (E = essential, D = desirable):**

**Experimental design:**

**Definition of experimental and control groups (E):**

- Control group (to which ΔCt was compared to): RNA from cells cultured conventionally (i.e., cells in medium with no gel and no scaffold)
- Experimental groups:
  - RNA from cells cultured in collagen scaffold
  - RNA from cells cultured in CMC-PEG gel
- Negative controls:
  - RNA from unseeded collagen scaffold
  - RNA from unseeded CMC-PEG gel
  - RNA from M2-10B4 (i.e., co-cultured murine cell line)
  - Nuclease-free water supplemented in Luna universal one-step RT-qPCR kit (New England Biolabs, Ipswich, MA, USA)
- Positive control:
  - RNA from monoculture of human primary cells acquired from a CLL patient

**Number within each group (E):**

- Cells of 15 patients (Supplemental Table S1) were cultured in three conditions (controls – conventionally; experimental groups – in collagen scaffold, in CMC-PEG gel)
- For each control group and experimental group:
  - 3 biological replicates for each patient and culture type
  - 3 RT-qPCR replicates for each RNA template
- For each negative and positive control:
  - 3 RT-qPCR replicates for each RNA template

**Assay carried out by core lab or investigator’s lab? (D):**

- By investigator’s lab, also with the equipment of Core Facility Genomics, CEITEC, Masaryk University Brno, Czech Republic

**Acknowledgement of author’s contributions (D):**

- M.D. was responsible for the patient selection and sample collection at the University Hospital Brno, K.P. selected the patient cohort based on diagnostic laboratory parameters and assisted with the experimental design, H.S. designed the experiments, carried out the cell culture, RNA isolation and RT-qPCR and prepared the data for statistical analysis, J.B. assisted with RNA isolation, primer design and evaluation, L.R. performed the statistical analysis. We would like to express our gratitude to the team of Vitezslav Bryja (namely Pavlina Janovska, and Milena Marakova; from the Institute of Experimental Biology, Faculty of Science, Masaryk University, Brno, Czech Republic) for providing *MYC* and *VCAM1* primers.

**Sample**

- **Description (E):** RNA isolated from human primary CLL cells co-cultured with murine M2-10B4 cell line either conventionally (in medium), or in biomaterials (i.e., collagen scaffolds or CMC-PEG gel)
  - Volume/mass of sample processed (D): For one RNA isolation, cells from one well of 96-well-plate were processed (i.e., ~8×10^5^ CLL cells)
  - Microdissection or macrodissection (E): Not applicable. Instead, neoplastic B cells were separated from peripheral blood with high purity (> 98 %) using Ficoll-Paque Plus (GE Healthcare, Uppsala, Sweden) coupled with RosetteSep human B cell enrichment cocktail and CD3+ cell depletion cocktail (StemCell Technologies, Vancouver, Canada). The purity of the cells was assessed by flow cytometry.
- **Processing procedure (E):** Vitally frozen.
  - If frozen – how and how quickly (E): Cells in 1 mL of 90% FBS and 10% dimethyl sulfoxide were pipetted into a cryo-tube (Nunc, Thermo Fisher Scientific, Roskilde, Denmark) and placed into a cryo-freezer filled with isopropyl alcohol (#LCC5100, National Lab, Mölln, Germany). The cryo-freezer was put into -80 °C for 24 to 72 hours; then, the cells were transferred to the liquid nitrogen (-196 °C).
  - If fixed – with what, how quickly (E): Not applicable.
- **Sample storage conditions and duration (E):**
  - At -196 °C for 0,4-14 years (median: 6,65 years)

**Nucleic acid extraction**

- **Procedure and/or instrumentation, name of kit and details of any modifications (E):** Described in the Materials and methods section of the main article text.
  - Source of additional reagents used (D): Not applicable.
- **Details of DNAse or RNAse treatment (E); contamination assessment (E):** None to reduce sample losses (amount of RNA extracted was too low). Compensated by use of primers spanning exon-exon junctions, where possible.
- **Nucleic acid quantification (E):** Performed with each RNA aliquot.
  - Instrument and method (E): Spectrophotometrically, NanoDrop 2000c (Thermo Fisher Scientific, Waltham, MA, USA).
  - Purity (A260/A280) (D): In raw data Zenodo file 10.5281/zenodo.11609920.
  - Yield (D): In raw data Zenodo file 10.5281/zenodo.11609920.
- **RNA integrity method/instrument (E):** Not performed due to low yields of RNA extraction.
  - RIN/RQI or Cq of 3’ and 5’ transcripts (E): Not evaluated.
  - Electrophoresis traces (D): Not evaluated.
- **Inhibition testing (Cq dilutions, spike or others, E):** Not performed.

**Reverse transcription (RT)**

- **Complete reaction conditions (E):** RT was performed in a single tube together with qPCR, as we used one-step RT-qPCR kit.
  - Amount of RNA and reaction volume (E): 1 μL of RNA, reaction volume: 10 μL
  - Priming oligonucleotide (if using GSP) and concentration (E): None.
  - Reverse transcriptase and concentration (E): WarmStart Luna Reverse Transcriptase supplemented in the kit in unknown concentration.
  - Temperature and time (E): 55 °C for 10 minutes.
  - Manufacturer of reagents and catalogue numbers (D): Luna universal one-step RT-qPCR kit (New England Biolabs, Ipswich, MA, USA; catalogue number NEB #E3005L).
  - Cqs with and without RT (D): Not performed.
- **Storage conditions of cDNA:** Not applicable – RT was done in the single tube together with qPCR which followed the RT step.

**qPCR target information**

- If multiplex, efficiency and LOD of each assay (E): Not multiplex.
- Sequence accession number (E): See the Supplemental Table S4.
- Location of amplicon (D): The primers annealed to the coding sequence of the respective transcripts (Table S4) and mostly spanned an exon-exon junction, except for primers for the *MYC* gene, in which the species-specific design spanning the exon-exon junction was not possible.
- Amplicon length (E): See the Supplemental Table S4.
- *In silico* specificity screen (BLAST, etc): Performed by Primer-BLAST (Ye et al., 2012). The primers were checked for unspecific targets in *Mus musculus* to avoid unspecific targets in the co-cultured murine cells, and *Bos taurus*, as collagen scaffolds were of a bovine origin. Primers were specific to input template as no other targets were found in selected database: Refseq mRNA (Organism limited to *Mus musculus*, *Homo sapiens*, *Bos taurus*). The primer specificity was experimentally verified using mouse RNA isolated from M2-10B4 cells, human RNA isolated from a CLL patient, RNA isolated from collagen scaffolds and CMC-PEG gel without any cells. Ten times higher input of murine RNA (100 ng) than human RNA (10 ng) was used for individual validation experiments as this corresponded to a ratio of RNA amount isolated from a portion of scaffold-/gel-cultured murine M2-10B4 cells or primary human CLL cells, respectively. Even with this difference in the input, the primers did not anneal to murine RNA and targeted human RNA (Fig. S1).
- Location of each primer by exon or intron (if applicable) (E): See the Supplemental Table S4.
- What splice variants are targeted (E): See the Supplemental Table S4.

**qPCR oligonucleotides**

- Primer sequences (E): See the Supplemental Table S4.
- Location and identity of any modifications (E): No modifications.
- Manufacturer of oligonucleotides (D): Generi Biotech, Hradec Králové, Czech Republic.
- Purification method (D): Standard purification at Generi Biotech, i.e., chemical residues were removed (excess of salts and protecting groups).

**qPCR protocol**

- Complete reaction conditions (E): Performed in a single tube together with RT, using the Luna universal one-step RT-qPCR kit (New England Biolabs, Ipswich, MA, USA; catalogue number NEB #E3005L).
  - Reaction volume and amount of cDNA/DNA (E): 10 μL of reaction volume, 1 μL of RNA as the input for RT which was immediately followed by qPCR.
  - Primer, (probe), Mg++ and dNTP concentrations (E), Buffer/kit identity and manufacturer (E): 10 μM of each primer (forward and reverse), Mg++ and dNTP concentrations unknown, as they were contained in the Luna Universal One-Step Reaction Mix (part of the kit mentioned above).
  - Exact chemical constitution of the buffer (D): Provided by the manufacturer for the Luna Universal One-Step Reaction Mix.
  - Additives (SYBR Green I, DMSO, etc.) (E): None.
- Manufacturer of the plates/tubes and catalog number (D): FrameStar 384-well skirted PCR plate, frosted wells, clear frame (#4ti-0387, 4titude, Surrey, United Kingdom)
- Complete thermocycling parameters (E): 55 °C for 10 minutes (RT), 95 °C for 1 minute (initial denaturation), 40 cycles of 95 °C for 10 seconds (denaturation) and 60 °C for 30 seconds (extension; plate read). A melt-curve step was added at the end (95 °C for 15 seconds, 60 °C for 60 seconds and 95 °C for 15 seconds).
- Reaction setup (manual/robotic) (D): Manual.
- Manufacturer of qPCR instrument (E): QuantStudio 12K Flex Real-Time PCR System (Applied Biosystems, Waltham, MA, USA).

**qPCR validation:**

- Evidence of optimization (from gradients) (D):
  - See the Supplemental Figure S1
- Specificity (gel, sequence, melt, or digest) (E):


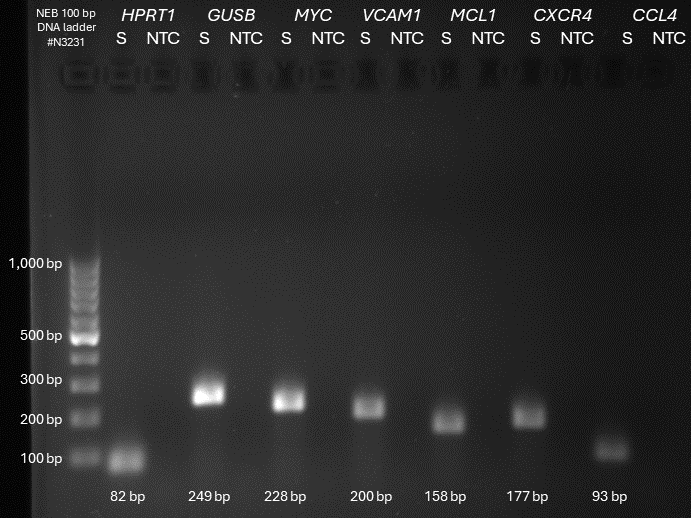


- - RT-qPCR products shown on an electrophoretic gel. All reactions yielded one product of expected size; no product was observed in negative controls. S – sample, NTC – no template control.
  - For melt profiles and raw image of the gel, see folder “Supplemental File 1” in the Zenodo file 10.5281/zenodo.11609920.
- For SYBR Green I, Cq of the NTC (E):
  - No amplified product during the qPCR.
- Standard curves with slopes and y-intercept (E):
  - PCR efficiency calculated from the slope (E), confidence interval for PCR efficiency or standard error (D), r2 of standard curve (E): See table below.
  - Linear dynamic range (E): Cq variation at lower limit (E), Confidence intervals throughout range (D), evidence for limit of detection (E): See table below and folders “Supplemental File 1/Standard curves” and “Supplemental File 1/Raw data and export files” in Zenodo file 10.5281/zenodo.11609920

| **Gene** | **Slope** | **y-intercept** | **PCR efficiency** | **R^2^** | **Error** | **Limit of detection** |
| --- | --- | --- | --- | --- | --- | --- |
| *HPRT1* | -3.295 | 31.407 | 101% | 0.990 | 0.103 | 0.01 ng |
| *GUSB* | -2.852 | 32.403 | 124% | 0.981 | 0.127 | 0.10 ng |
| *MYC* | -2.807 | 30.633 | 127% | 0.944 | 0.197 | 0.01 ng |
| *VCAM1* | -1.026 | 36.938 | 844% | 0.665 | 0.364 | 10.00 ng |
| *MCL1* | -3.299 | 29.698 | 101% | 0.986 | 0.113 | 0.01 ng |
| *CXCR4* | -3.050 | 32.059 | 113% | 0.960 | 0.188 | 0.10 ng |
| *CCL4* | -3.022 | 30.121 | 114% | 0.991 | 0.079 | 0.05 ng |

- If multiplex, efficiency and LOD of each assay (E): Not Applicable

**Data analysis:**

- qPCR analysis program (source, version) (E): Data were analyzed in the QuantStudio 12K Flex Software, v1.4.
  - Cq method determination (E): A PCR cycle number at with a sample’s reaction curve intersects a threshold line. If one gene was analyzed in two plates, the threshold line was adjusted to equal Cq in the positive control for each plate.
  - Outlier identification and disposition (E): Data cleaned manually after plotting the graphs in GraphPad software. If outlier was removed, the value was replaced by the median of the remaining values in the same group. If all the sample replicates (technical/biological) had outlying values compared to the rest of the dataset (i.e., orders of magnitude), they were removed from the dataset.
- Results of NTCs (E): Negative, see Supplemental Figure 1.
- Justification of number and choice of reference genes (E):
  - Two reference genes, their number and selection were chosen based on this publication: Valceckiene V, Kontenyte R, Jakubauskas A, Griskevicius L. Selection of reference genes for quantitative polymerase chain reaction studies in purified B cells from B cell chronic lymphocytic leukaemia patients [published correction appears in Br J Haematol. 2011 Jul;154(1):157]. Br J Haematol. 2010;151(3):232-238. doi:10.1111/j.1365-2141.2010.08363.x
- Description of normalization method (E):
  - ΔCt method was used: Ct of each sample (average of the three technical triplicates) was subtracted by Ct of two reference genes, *HPRT1* and *GUSB* (each time average of the three technical replicates)*.* These two differences were then averaged, resulting in ΔCt.
  - ΔCt value of each sample cultured in 3D (collagen scaffolds, CMC-PEG gel) was then subtracted by corresponding ΔCt value of the sample cultured conventionally, resulting in ΔΔCt.
- Number and concordance of biological replicates (D):
  - 3 biological replicates; their variability is plotted in Supplemental Figure S4.
- Number and stage (RT or qPCR) of technical replicates (D):
  - 3 technical replicates for one-step RT-qPCR reaction.
- Repeatability (intra-assay variation) (E): See folder “Supplemental File 1/Raw data and export files” in the Zenodo file 10.5281/zenodo.11609920.
- Statistical methods for result significance (E): See the full article (Materials and methods - Statistical analysis). Statistical analysis was performed using the limma method (linear models), which was primarily developed for evaluating differential expression across different sample groups. All parameters of the experiment (i.e., type of culture, IGHV status, *TP53* mutational status, *NOTCH1* mutational status, patient ID) were inserted into the design matrix of the used linear model, and then statistically significant differences between individual groups were tested.
- Software (source, version) (E): R, version 4.1.1. R Core Team (2021). R: A language and environment for statistical computing. R Foundation for Statistical Computing, Vienna, Austria. URL https://www.R-project.org/.
