# Supplementary material for: In vitro culture of leukemic cells in collagen scaffolds and carboxymethyl cellulose-polyethylene glycol gel
Source: PeerJ. 2024 Dec 6;12:e18637. doi: 10.7717/peerj.18637 (PMC11627079; doi:10.7717/peerj.18637)
Supplement: Supplemental Information 7 — M – male, F – female, Mut – mutated, Unmut – unmutated, WT – wild type, NA – not available. [file peerj-12-18637-s007.docx]

**Table S1**: Characteristics of patients and their CLL cells used in experiments. M – male, F – female, Mut – mutated, Unmut – unmutated, WT – wild type, NA – not available.

| Group | ID | Sex | Age at diagnosis (years) | Initial viability of cells (%) | IGHV | *TP53* | *NOTCH1* | FISH results | Previously treated | Included in article results |
| --- | --- | --- | --- | --- | --- | --- | --- | --- | --- | --- |
| None | Pt01 | M | 65 | 98.4 | Unmut | WT | NA | +12 | Yes | Fig. 4, 6, S1, S3 |
|  | Pt02 | M | 67 | 92.3 | Mut | WT | NA | del13q14 | Yes | Fig. 5, S2 |
|  | Pt03 | M | 59 | 98.1 | Mut | Mut | NA | del13q14 | Yes |  |
|  | Pt04 | F | 68 | 93.3 | Mut | WT | NA | del13q14 | Yes |  |
|  | Pt05 | M | 55 | 83.9 | Mut | WT | WT | +12, del13q14 | Yes |  |
|  | Pt06 | F | 63 | 85.4 | Mut | Mut | Mut | del13q14 | Yes |  |
|  | Pt07 | M | 58 | 86.2 | Unmut | WT | WT | del11q, del13q14 | Yes |  |
| Gr1 | Pt08 | M | 82 | 90.5 | Unmut | Mut | Mut | del17p, del13q14 | No | Fig. 7-9, S4, S5; Tables 2, 3 |
|  | Pt09 | F | 48 | 94.8 | Unmut | Mut | Mut | del17p, del11q, del13q14 | Yes |  |
|  | Pt10 | F | 46 | 93.8 | Unmut | Mut | Mut | del17p, del11q, del13q14 | Yes |  |
| Gr2 | Pt11 | M | 30 | 95.1 | Unmut | Mut | WT | del17p | No |  |
|  | Pt12 | M | 54 | 98.2 | Unmut | Mut | WT | Negative | Yes |  |
|  | Pt13 | M | 47 | 77.9 | Unmut | Mut | WT | del17p | Yes |  |
| Gr3 | Pt14 | M | 52 | 96.8 | Unmut | WT | Mut | Negative | No |  |
|  | Pt15 | F | 82 | 98.4 | Unmut | WT | Mut | del13q14 | Yes |  |
|  | Pt16 | M | 60 | 88.6 | Unmut | WT | Mut | del13q14 | Yes |  |
| Gr4 | Pt17 | M | 59 | 86.2 | Unmut | WT | WT | Negative | No |  |
|  | Pt18 | F | 50 | 87.3 | Unmut | WT | WT | del11q, del13q14 | No |  |
|  | Pt19 | F | 47 | 94.0 | Unmut | WT | WT | Negative | No |  |
| Gr5 | Pt20 | M | 73 | 95.9 | Mut | WT | WT | Negative | No |  |
|  | Pt21 | F | 62 | 95.2 | Mut | WT | WT | del13q14 | No |  |
|  | Pt22 | M | 63 | 96.3 | Mut | WT | WT | del13q14 | No |  |
| None | Pt23 | M | 55 | 75.7 | Unmut | WT | NA | NA | No | Fig. 10 |
|  | Pt24 | F | 76 | 89.4 | Unmut | WT | NA | NA | No |  |
|  | Pt25 | M | 62 | 78.5 | Unmut | Mut | NA | del13q14 | No |  |
|  | Pt26 | M | 52 | 83.8 | Mut | WT | NA | del13q14 | Yes |  |
|  | Pt27 | F | 52 | 83.3 | Mut | WT | NA | NA | No |  |
