# Supplementary material for: In vitro culture of leukemic cells in collagen scaffolds and carboxymethyl cellulose-polyethylene glycol gel
Source: PeerJ. 2024 Dec 6;12:e18637. doi: 10.7717/peerj.18637 (PMC11627079; doi:10.7717/peerj.18637)
Supplement: Supplemental Information 8 [file peerj-12-18637-s008.docx]

**Table S2:** Media used for culture of cells (Biosera, Nuaille, France).

| **Cells** | **Medium** | **Supplements included in the medium** | **Catalogue number** |
| --- | --- | --- | --- |
| HS-5 | Dulbecco’s Modified Eagle Medium (DMEM) | 4.5 g/L (*w/v*) glucose, stable glutamine and sodium pyruvate | LM-D1110/500 |
| M2-10B4 monoculture |  |  |  |
| MEC-1 | Iscove’s Modified Dulbecco’s Medium (IMDM) | Stable glutamine and 25 mM HEPES | LM-I1091/500 |
| HG-3 | RPMI 1640 | Stable glutamine and 25 mM HEPES | LM-R1638/500 |
| Primary CLL cells |  |  |  |
| M2-10B4 intended for co-culture |  |  |  |
