# Supplementary material for: In vitro culture of leukemic cells in collagen scaffolds and carboxymethyl cellulose-polyethylene glycol gel
Source: PeerJ. 2024 Dec 6;12:e18637. doi: 10.7717/peerj.18637 (PMC11627079; doi:10.7717/peerj.18637)
Supplement: Supplemental Information 9 [file peerj-12-18637-s009.docx]

**Table S3**: Series of ImageJ commands used for counting viability of one cell type in a co-culutre of two cell types.

| Step | Commands |
| --- | --- |
| 1) Converting to binary image | “Image” → “Adjust” → “Threshold” (Otsu’s method), “Red” 🗸, “Dark Background” 🗸; proceeding without confirmation |
| 2) Selecting foreground | “Edit” → “Selection” → “Create selection” |
| 3) Creating region of interest (ROI) | ROI added to the “ROI Manager” by pressing “T”; ROI renamed |
| 4) Steps 1-3 repeated for each relevant channel. | |
| 5) Creating overlap of ROIs | Desired ROIs selected in ROI Manager → “More” → “AND” → overlap added to the “ROI Manager” by pressing “T”; ROI (overlap) renamed |
| 6) Setting the measurement parameters | “Analyze” → “Set measurements” → “Area” 🗸, “Display Label” 🗸 → “OK”; |
| 7) Measuring the area of each ROI | For each desired combination of a channel and a ROI, a window with the corresponding channel was selected and then the ROI was chosen in “ROI Manager”.  “ROI Manager”: “Measure”  The “Total Area” value was used for subsequent calculation. |
| 8) Measuring the viability of one cell type in co-culture of two cell types | Example:  Channel 1 = All CLL cells  Channel 2 = All dead cells (dead M2-10B4 cells, dead CLL cells)  Channel 3 = All M2-10B4 cells  Channel 4 = Transmission channel  To count viability of CLL cells, steps 1-3 were performed for channel 1 and 2. Overlap was performed with ROIs from channel 1 and 2 (i.e., overlap includes only dead CLL cells). While selecting the Channel 1 window, ROI created from channel 1 was measured (Total Area 1 = number of all CLL cells). Then, in the Channel 1 window, ROI created from overlap was measured (Total Area 2 = number of dead CLL cells).  Viability of CLL cells = 100%-((Total Area 2/Total Area 1)*100)) |
