# Supplementary material for: In vitro culture of leukemic cells in collagen scaffolds and carboxymethyl cellulose-polyethylene glycol gel
Source: PeerJ. 2024 Dec 6;12:e18637. doi: 10.7717/peerj.18637 (PMC11627079; doi:10.7717/peerj.18637)
Supplement: Supplemental Information 10 [file peerj-12-18637-s010.docx]

**Table S4:** **Validated human-specific primers used for qPCR** **with no targets in *Mus musculus* and *Bos taurus***.

| Gene | Transcript variants | GenBank Number | Exon-exon junction | Target exon(s) | Forward Primer (5’ 🡪 3’) | Reverse Primer (5’ 🡪 3’) | Product Length (bp) |
| --- | --- | --- | --- | --- | --- | --- | --- |
| *HPRT1* | 1 | NM_000194.3 | Yes | e6-e8 | CAAAGATGGTCAAGGTCGCA | TCAAATCCAACAAAGTCTGGC | 82 |
| *GUSB* | 1 | NM_000181.4 | Yes | e3-e5 | CCGGCTCCGAATCACTATCG | TGGTAATTCACCAGCCCACT | 249 |
| *MYC* | 1, 2 | NM_002467.6,  NM_001354870.1 | No | e3 | TGTCCCAAGCACTCCTAAGC | CCAGCTGATTCCTACCGTCG | 228 |
| *VCAM1* | 1 | NM_001078.4 | Yes | e1-e2 | ATTTCACTCCGCGGTATCTGC | GCTTGAGAAGCTGCAAACATTA | 200 |
| *MCL1* | 2 | NM_182763.3 | Yes | e1b-e3 | TCTCTCGGTACCTTCGGGAG | TCCACAAACCCATCCTTGGAA | 158 |
| *CXCR4* | 4 | NM_001348059.2 | Yes | e1-e2 | GCTTGGGGGAGGAGATATACA | TGACCAATCCATTGCCCACA | 177 |
| *CCL4* | 1 | NM_002984.4 | Yes | e2-e3 | GCTGTGGTATTCCAAACCAAAAGA | GTTCAGTTCCAGGTCATACACG | 93 |
